# The renoprotective activity of amikacin–gamma-amino butyric acid–chitosan nanoparticles: a comparative study

**DOI:** 10.1007/s10787-024-01464-5

**Published:** 2024-04-25

**Authors:** Neveen Madbouly, Adham Ooda, Ahmed Nabil, Areej Nasser, Esraa Ahmed, Fatma Ali, Fatma Mohamed, Habiba Faried, Mai Badran, Mariam Ahmed, Mariam Ibrahim, Mariam Rasmy, Martina Saleeb, Vereena Riad, Yousr Ibrahim, Alyaa Farid

**Affiliations:** 1 Zoology Department, Faculty of Science, Cairo University, Giza, Egypt; 2 Biotechnology/Biomolecular Chemistry Program, Faculty of Science, Cairo University, Giza, Egypt; 3 Biotechnology Department, Faculty of Science, Cairo University, Giza, Egypt

**Keywords:** Amikacin, Gamma-amino butyric acid, Chitosan, Nanoparticles, Nephrotoxicity, Inflammatory cytokines

## Abstract

The development of nanoparticles (NPs) with active components with upgraded stability, and prolonged release helps in enhanced tissue regeneration. In addition, NPs are feasible strategy to boost antibiotic effectiveness and reduce drug side effects. Our study focuses on the use of amikacin (AMK) and gamma amino butyric acid (GABA) unloaded combinations or loaded on chitosan nanoparticles (CSNPs) for kidney protection. The AMK–GABA–CSNPs were prepared with the ionic gelation method, the morphology was studied using transmission electron microscopy (TEM), zetasizer and the Fourier transform-infrared spectroscopy (FT-IR) spectrum of the synthesized NPs was observed. The average size of AMK–GABA–CSNPs was 77.5 ± 16.5 nm. Zeta potential was + 38.94 ± 2.65 mV. AMK–GABA–CSNPs revealed significant in vitro antioxidant, anti-coagulation, non-hemolytic properties and good cell compatibility. To compare the effects of the unloaded AMK–GABA combination and AMK–GABA–CSNPs on the renal tissue, 42 healthy Sprague–Dawley rats were divided into seven groups. G1: normal control (NC), normal saline; G2: low-dose nephrotoxic group (LDN), AMK (20 mg/kg/day; i.p.); G3: unloaded AMK (20 mg/kg/day; i.p.) and GABA (50 mg/kg/day; i.p.); G4: AMK–GABA–CSNPs (20 mg/kg/day; i.p.); G5: high-dose nephrotoxic group (HDN), AMK (30 mg/kg/day; i.p.); G6: unloaded AMK (30 mg/kg/day; i.p.) and GABA (50 mg/kg/day; i.p.) and G7: AMK–GABA–CSNPs (30 mg/kg/day; i.p.). The results showed that AMK–GABA–CSNPs formulation is superior to unloaded AMK–GABA combination as it ameliorated kidney functions, oxidative stress and displayed a significant homeostatic role via suppression of inflammatory cytokines of Th1, Th2 and Th17 types. Hence, AMK–GABA–CSNPs could afford a potential nano-based therapeutic formula for the management of AMK-nephrotoxicity.

## Introduction

Nephrotoxicity could be induced by various substances in a variety of patterns and mechanisms. Tubular necrosis, interstitial nephritis, crystal nephropathy, angiopathy, changes in intraglomerular hemodynamics, rhabdomyolysis, and fibrosis are the most common signs of drug-induced nephrotoxicity (DIN), which can lead to renal failure (Ghane Shahrbaf and Assadi 2015; Gao et al. 2021). DIN reduces the efficacy of drugs and complicates the treatment of serious cancers (Kintzel 2001), renal transplantation (de Mattos et al. 2000) and several metabolic disorders (Raza and Naureen 2020).

Among the drugs that have a nephrotoxic effect is amikacin (AMK) (Azırak 2023). AMK is a semisynthetic, broad-spectrum aminoglycoside antibiotic derived from kanamycin A. Despite its strong antibacterial activity, low cost, rapid onset of action, synergy with beta-lactam antibiotics, and low resistance, nephrotoxicity and ototoxicity limit AMK clinical applicability (Wargo and Edwards 2014). Multiple pathophysiological effects, including inflammation, inhibition of specific transporters, induction of oxidative stress and apoptosis, vascular alterations, contribute to AMK-induced renal toxicity (Prajapati and Singha 2010; Hlail et al. 2020).

Gamma-aminobutyric acid (GABA) is a non-protein amino acid that is primarily found in the central nervous system especially the brain, where it functions as a significant inhibitory neurotransmitter (Olsen and Betz 2006). Notable GABA biological activities include anti-diabetes, anti-hypertension, anti-cancer, antioxidant, antimicrobial, anti-allergenic and anti-inflammatory properties (Han et al. 2007; Prud’homme et al. 2013; Ngo and Vo 2019). In addition, GABA was reported to protect the intestine, liver and kidney from toxin-induced injury (Rashmi et al. 2018). GABA was demonstrated to be capable of trapping reactive intermediates during lipid peroxidation (Deng et al. 2010).

Nanoparticles (NPs) are remarkable drug delivery systems which are utilized due to their stability, unique properties (e.g., biodegradability, biocompatibility, and nontoxicity) in comparison to bulkier equivalents (Ravishankar and Jamuna 2011; Divya and Jisha 2018). Chitosan nanoparticles (CSNPs) possess the properties of CS and NPs, including nontoxicity, biodegradability, antimicrobial properties, interface effects, small size, and quantum size effects (Ingle et al. 2008). CSNPs can also deliver medicines or macromolecules to targeted sites via controlled release and modifying their pharmacokinetics (López-León et al. 2005; Shi et al. 2011, 2012; Perera and Rajapakse 2013). This study aimed to synthesize and characterize AMK–GABA–CSNPs, then evaluate their intraperitoneal (i.p.) injection in Sprague–Dawley rats for effective administration with minimum side effects. We compared the renoprotective effects of unloaded AMK–GABA combination with that of AMK–GABA–CSNPs.

## Methods

### Treatments and chemicals

AMK-sulfate injection: (Amikin® 500 mg, EIPICO pharmaceuticals Co., 10Th of Ramadan City, Ash Sharqiyah, Egypt). GABA: (Sigma Chemical Co., St. Louis, USA). CS: (low molecular weight, deacetylation degree: 93%) (Oxford Inc., India). All chemicals were provided from Sigma Aldrich (St. Louis, MO).

## Preparation of AMK–GABA–CSNPs

CSNPs were synthesized based on ionic gelation method with sodium tripolyphosphate (TPP) as a cross-linking agent (Reddy and Damodharan 2022). 0.5 g CS powder was added to 65 ml 1% acetic acid solution on a magnetic stirrer and was filtered through a Millipore membrane to get rid of any contaminants. 0.25 g TPP powder was dissolved in 100 ml distilled water. To the TTP solution, AMK (20 mg/ml) and GABA (50 mg/ml) were added and dissolved completely. The TTP–AMK–GABA solution is gradually added dropwise to the CS solution that is being stirred by a magnetic device. Overnight stirring is done with this solution. The synthesized NPs were separated by centrifuging the resulting suspension for 10 min at a speed of 10,000 RPM. NPs were washed thoroughly three times with distilled water, lyophilized and stored in an airtight container.

### Characterization of AMK–GABA–CSNPs

#### Morphological studies

Using transmission electron microscope (TEM), the shape of the produced NPs was determined. Particle size (PS) of the formulated AMK–GABA–CSNPs was determined by the dynamic light scattering technique, and zeta potential (ZP) was measured (Zetasizer Nano ZS90; Malvern Instruments Limited, Malvern, UK).

#### Fourier transform-infrared spectroscopy (FT-IR)

At room temperature, the interaction between CS, AMK, GABA, and AMK–GABA–CSNPs components was studied using FT-IR (Spectrum Two, USA). After being combined with potassium bromide, the freeze-dried samples were compressed. The wavenumber range for all spectra was 4000–400 cm^−1^, with a resolution of 4 cm^−1^.

### In vitro studies

#### In vitro antioxidant assay

Using 1,1-diphenyl-2-picryl hydrazyl (DPPH) radical scavenging assay, the antioxidant property of the formulated AMK–GABA–CSNPs was determined (Souza et al. 2012). AMK–GABA–CSNPs were prepared at serial dilutions (1.95, 3.9, 7.8, 15.62, 31.25, 62.5, 125, 250, 500, 1000 µg/ml) were carefully disturbed and mixed with 0.1 mM DPPH. The solution was incubated at 25 °C in the dark for 30 min. The decline in concentrations of DPPH was observed by evaluating absorbance at 517 nm. Finally, using ascorbic acid as the standard reference, the antioxidant activity of AMK–GABA–CSNPs was determined in three samples. The DPPH scavenging ability was expressed in percentage (%) as follows:

$${\text{DPPH scavenging assay \% }} = \frac{{{\mathrm{Control~absorbance}} - {\text{}}\left( {{\text{sample absorbance}} - {\text{blank absorbance}}} \right)}}{{{\mathrm{Control}}~{\mathrm{absorbance}}}} \times ~100.$$

#### In vitro coagulation assay

The anticoagulating activity of AMK–GABA–CSNPs was assessed according to Jesus et al. (2020). The two blood coagulation pathways, the partial thromboplastin time (PTT) and the prothrombin time (PT) were evaluated separately, with heparin serving as the control. The plasma was obtained by centrifuging the blood at 2500*g* for 10 min after collecting the blood in sodium citrate tubes. 900 µL of rat plasma was treated with various doses of AMK–GABA–CSNPs or heparin (25, 50, and 75 µg/ml) (suspended in saline), for 30 min at 37 °C. The procedure was repeated by incubating plasma with CSNPs. The test was performed in three samples, and the clotting time was recorded using an ACL 200 coagulation analyzer (Diamond Diagnostics Inc., Bedford, MA, USA).

#### In vitro hemolysis assay

AMK–GABA–CSNP hemolytic potential was assessed using a membrane stabilization experiment based on Daraba et al. (2020). Fresh blood from a healthy rat was taken for these tests. 5 mL of heparinized blood was centrifuged at 3000 rpm for 5 min and washed multiple times with normal saline solution to remove serum and obtain erythrocytes. The purified erythrocytes were then resuspended in 40% v/v isotonic saline solution. To the erythrocyte suspension, 5 mL of AMK–GABA–CSNPs suspension in distilled water (hypotonic solution) at various concentrations (100, 200, 400, 600, 800, and 1000 g/ml) were added. By adding equivalent amounts (5 mL) of indomethacin and normal saline solution, respectively, positive (100% lysis inhibition) and negative (0% lysis inhibition) control samples were generated. The samples were incubated at 37 °C for 60 min. Following the incubation period, the samples were centrifuged at 1500 rpm for 3 min. At 540 nm, the released hemoglobin in the supernatant was measured using a spectrophotometer. Every sample was examined in triplicate. Hemolysis inhibition percentage (%) was estimated using the formula:

$$\mathrm{Hemolysis inhibition percentage }\left(\mathrm{\%}\right)=1- \frac{\mathrm{OD sample }-\mathrm{ OD negative control}}{\mathrm{OD positive control }-\mathrm{ OD negative control}} {\mathrm{x}} 100.$$

#### In vitro cytotoxicity (MTT) assay

The cytotoxicity was determined using the 3-(4,5-dimethylthiazol-2-yl)-2,5-diphenyltetrazolium bromide (MTT) assay established by Jesus et al. (2020). Human intestinal Caco-2 cell line, was obtained from The National Research Centre, Cairo, Egypt. Cells were seeded at high density (100 µl/well, 10^5^ cells) in a 96-well polystyrene-coated plate (flat bottomed). Caco-2 cells were propagated for 48 h in 95% air, 100% relative humidity, and 5% CO_2_ at 37 °C using Eagle minimal essential medium (EMEM) supplemented with 20% fetal bovine serum (FBS) to establish of cell monolayers. Three samples of AMK–GABA–CSNPs and CSNPs at variant concentrations (31.25, 62.5, 125, 250, 500, and 1000 µg/ml) in RPMI medium were added to the cultured cells and incubated for 24 h. MTT reagent (20 µl) at a concentration of 5 mg/ml was added and incubated for 4 h at 37 °C. The formazan crystals resulted during the incubation period were dissolved by adding 100 μL DMSO to each well with gentle dissolution. The absorbance was read in micro plate reader at 570 nm; the medium served as a control. The cell viability percentage was evaluated with respect to control (taken as 100%). The IC50 (the concentration of drug that displayed 50% cell survival) was calculated according to Wang et al. (2008). The cell viability percentage (%) was calculated according to the following formula:

$${\text{Cell viability}}\left( \% \right)\, = \,{\mathrm{absorbance}}\left( {{\mathrm{test}}} \right)/{\mathrm{absorbance}}\left( {{\mathrm{control}}} \right)\, \times \,{\mathrm{1}}00.$$

### In vivo studies

#### Animals

Male, 180–200 g, 10–12 weeks old Sprague–Dawley rats were used. Rats were bought from the National Research Centre’s animal house colony in Cairo, Egypt. To rule out any underlying infection, animals were housed in adequate laboratory settings for a week before the beginning of the investigation. Standard pellet food and water were provided to the animals.

#### Ethical approval

The experimental protocol was approved by the Faculty of Science’s Institutional Animal Care and Use Committee (IACUC) with consent number CUIF6922.

#### Experimental design

Forty-two Sprague–Dawley rats were divided into seven groups (*n* = 6/group) and treated as follows: G1: normal control (NC), received normal saline; G2: low-dose nephrotoxic group (LDN), received AMK (20 mg/kg/day; i.p.); G3: received unloaded AMK (20 mg/kg/day; i.p.) and GABA (50 mg/kg/day; i.p.); G4: received AMK–GABA–CSNPs (20 mg/kg/day; i.p.); G5: high-dose nephrotoxic group (HDN), received AMK (30 mg/kg/day; i.p.); G6: received unloaded AMK (30 mg/kg/day; i.p.) and GABA (50 mg/kg/day; i.p.) and G7: received AMK–GABA–CSNPs (30 mg/kg/day; i.p.), injections were continued for 21 days and rats were euthanized on the 22nd day.

#### Serum preparation and kidney functions

All animals were kept under anesthesia before having blood samples taken from their retro-orbital venous plexus. The blood was left to clot at room temperature. Serum was prepared from the blood after centrifuging it at 3000 rpm for 15 min. Serum was collected and kept at −20 °C to assess kidney functions. Blood urea nitrogen (BUN) (Marsh et al. 1965), creatinine (CRE) (Bonses and Taussky 1945), and uric acid (UA) (Newman and Price 1994) were assessed.

#### Kidney homogenate

The right kidney was used to prepare the renal tissue homogenate. Rapid kidney exclusion, washing in a physiological saline solution, and removal of surrounding fatty tissues. In 10 ml of phosphate buffered saline, 1 g of tissue was homogenized and centrifuged at 6000 rpm for 10 min (Ashry et al. 2021). The supernatant was collected and kept at −20 °C to perform biochemical and immunological assays.

#### In vivo antioxidant assays

Malondialdehyde (MDA) content, superoxide dismutase (SOD) activity and reduced glutathione (GSH) content, were determined in renal homogenate supernatant using commercially available colorimetric assay kits (Elabscience, Texas, USA) according to the suppliers’ protocol (Cat Number: E-BC-K025-M, E-BC-K019-S and E-BC-K030-M, respectively).

MDA content, in kidney homogenate supernatant, was measured based on thiobarbituric acid (TBA) method (Placer et al 1966). In this method, MDA reacts with TBA to form MDA-(TBA)_2_, a red-colored adduct, which has a maximum absorption peak at 532 nm. To measure MDA level, For each sample, 0.02 mL kidney homogenate supernatant, 0.02 mL of clarificant, 0.6 mL of acid application solution, 0.2 mL of chromogenic application solution into standard tubes and sample tubes and 0.2 mL of 50% acetic acid to the control tubes were mixed and placed in boiling water for 40 min. After cooling the samples were centrifuged at 9569*g* for 10 min. Finally 0.25 mL of each supernatant was transferred to the microplate and the optical density of samples was measured at 535 nm.

SOD activity was determined according to hydroxylamine method (Crouch et al. 1981) which based on the ability of SOD to specifically inhibit superoxide anion free radical. SOD inhibit the reduction of nitroblue tetrazolium (NBT) to blue formazan from hydroxylamine in alkaline solution. The NBT reduction was spectrophotometrically measured at 560 nm. Briefly, 1 mL of buffer working solution was added to 1 ml of diluted kidney supernatant. The samples were mixed well and 0.1 mL of nitrosogenic agent, 0.1 mL of substrate solution, 0.1 mL of enzyme stock working solution were added successively into each tube. The reaction components were mixed with a vortex and incubated at 37 ℃ for 40 min finally 2 mL of chromogenic agent was added, mixed and left in room temperature for 10 min. The absorbance was measured at 505 nm and the SOD activity was then calculated according to the manufacturer’s instruction.

GSH level was estimated following the method described by Ellman (1959). In this method, thiols react with 5,5′-dithiobis-(2-nitrobenzoic acid) (DTNB), to give 2-nitro-5-thiobenzoate (TNB −), which ionizes to the TNB2 − dianion in water at neutral and alkaline pH to form a yellow complex which is then detected by colorimetric assay at 405 nm. To evaluate GSH level in samples, 100 μL of kidney supernatant was mixed with 25 μL of DTNB solution and 100 μL of phosphate solution. Samples were mixed well and incubated for 5 min at room temperature and the TNB2− development was distinguished in a spectrophotometer by measuring the absorbance peak at 405 nm.

### Inflammatory cytokines

Rat ELISA kits (Abcam, Cambridge, UK) were used to measure IL-1β (Cat Number: ab100768), IL-4 (Cat Number: ab100771), IL-6 (Cat Number: ab100772), IL-17 (Cat Number: ab119536), IL-18 (Cat Number: ab213909), TNF-α (Cat Number: ab100785) and IFN-γ (Cat Number: ab46107) in kidney homogenates from the different groups according to the manufacturer’s instructions.

#### Histopathological studies

The left kidney from each rat in all groups was preserved in 10% formalin solution, dehydrated in graded alcohol and then sectioned in paraffin wax at 4 µm thickness. Hematoxylin and eosin (H&E) staining was applied to detect general histopathological alterations. Masson's trichrome was used for semiquantitative examination of collagen and interstitial fibrosis (Macsween 1977). The accumulated collagen in the interstitial area appeared light blue under the microscope. Each kidney section was examined in ten randomly selected fields under × 400 Olympus CX41 microscope (Olympus Corporation, Shinjuku, Tokyo, Japan) in a blinded manner by two experienced pathologists in Department of Pathology, Al-Azhar Faculty of Medicine, Cairo, Egypt. Image Pro Plus (IPP) 6.0 software (Media Cybernetics, Inc., Rockville, USA) was used to quantify the percentage of tubulointerstitial fibrotic area in ten randomly examined fields from each section, and the average percentage of renal fibrotic area for each section was calculated.

### Statistical analysis

Each in vitro assay was performed in triplicate from different samples, data were presented as mean ± SEM. All continuous variables were cheeked for normality to ensure uniformity of concentration via the Kolmogorov–Smirnov test of normality. Continuous data (e.g., BUN, CRE, UA, MDA, SOD, GSH, IL-1β, IL-4, IL-6, IL-17, IL-18, TNF-α and IFN-γ) are presented as (mean ± SEM). One-way ANOVA, with which multiple of comparisons post hoc Tukeyʼs test was applied on all studied groups using The Statistical Package for Social Services version 22 (SPSS) (IBM, Inc., Chicago, IL, USA). Alternatively, Mann–Whitney U test was used for non-parametric continuous variables (%interstitial fibrosis). The difference between the groups was deemed statistically significant if (*P* < 0.05).

## Results

### Morphology study

**Fig. 1:**
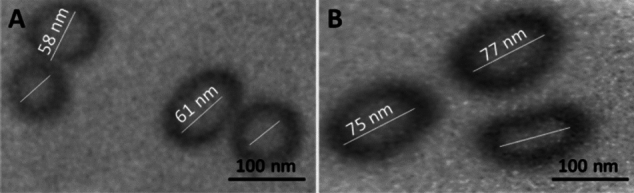
Transmission electron microscopy (TEM) images of **A** chitosan nanoparticles (CSNPs) and **B** amikacin–GABA chitosan nanoparticles (AMK–GABA–CSNPs), magnification of 10^5^X

The TEM analyzed images of CSNPs (Fig. 1A) and AMK–GABA–CSNPs (Fig. 1B) showed the typical spherical and uniform morphology of nanoparticles, which were well dispersed and separated from each other. The particle size of AMK–GABA–CSNPs ranged from 77.5 ± 16.5 nm and the ZP was + 38.94 ± 2.65 mV.

### Fourier transform‑infrared (FT-IR) analysis

**Fig. 2:**
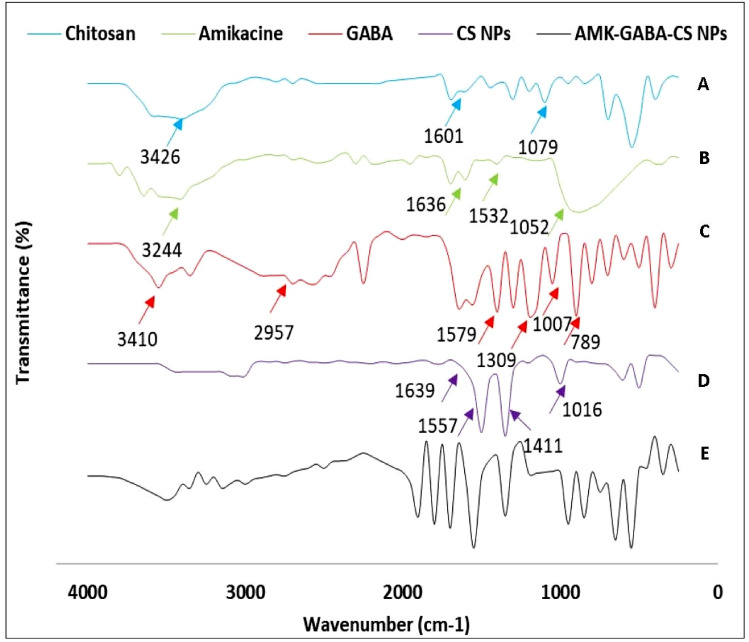
FTIR spectra of **A** chitosan, **B** amikacin, **C** GABA, **D** chitosan nanoparticles (CSNPs) and **E** AMK–GABA–CSNPs

FTIR analysis of CS, AMK, GABA, CSNPs and AMK–GABA–CSNPs was conducted to confirm the success of the synthesis process. FTIR spectra of CS (Fig. 2A) showed characteristic peaks at 3426 cm^−1^ (–OH and –NH2), 1601 cm^−1^ –NH2), and 1076 cm^−1^ (C–O–C). While AMK (Fig. 2B) showed 3244 cm^−1^ (–OH), 1636 cm^−1^ (N–H), 1532 cm^−1^ (–CN–), 1277 cm^−1^ and 1052 cm^−1^ (C–O). GABA (Fig. 2C) showed characteristic peaks at 3410 cm^−1^ (–OH), 2957 cm^−1^ (–CH_2_), 1579 cm^−1^ (NH_3_^+^), 1309 cm^−1^ (–CH_2_), 1007 cm^−1^ (–NH_3_) and 789 cm^−1^ (COO^−^). CSNPs (Fig. 2D) showed the typical peaks at 1639 cm^−1^ (–CONH2), 1557 cm^−1^ (–NH2), 1411 cm^−1^ (–CH2) and 1016 cm^−1^ (P = O). Finally, AMK–GABA–CSNPs (Fig. 2E) showed the CSNPs spectrum superimposed with the typical AMK and GABA peaks.

### Antioxidant activity of AMK–GABA–CSNPs

**Fig. 3:**
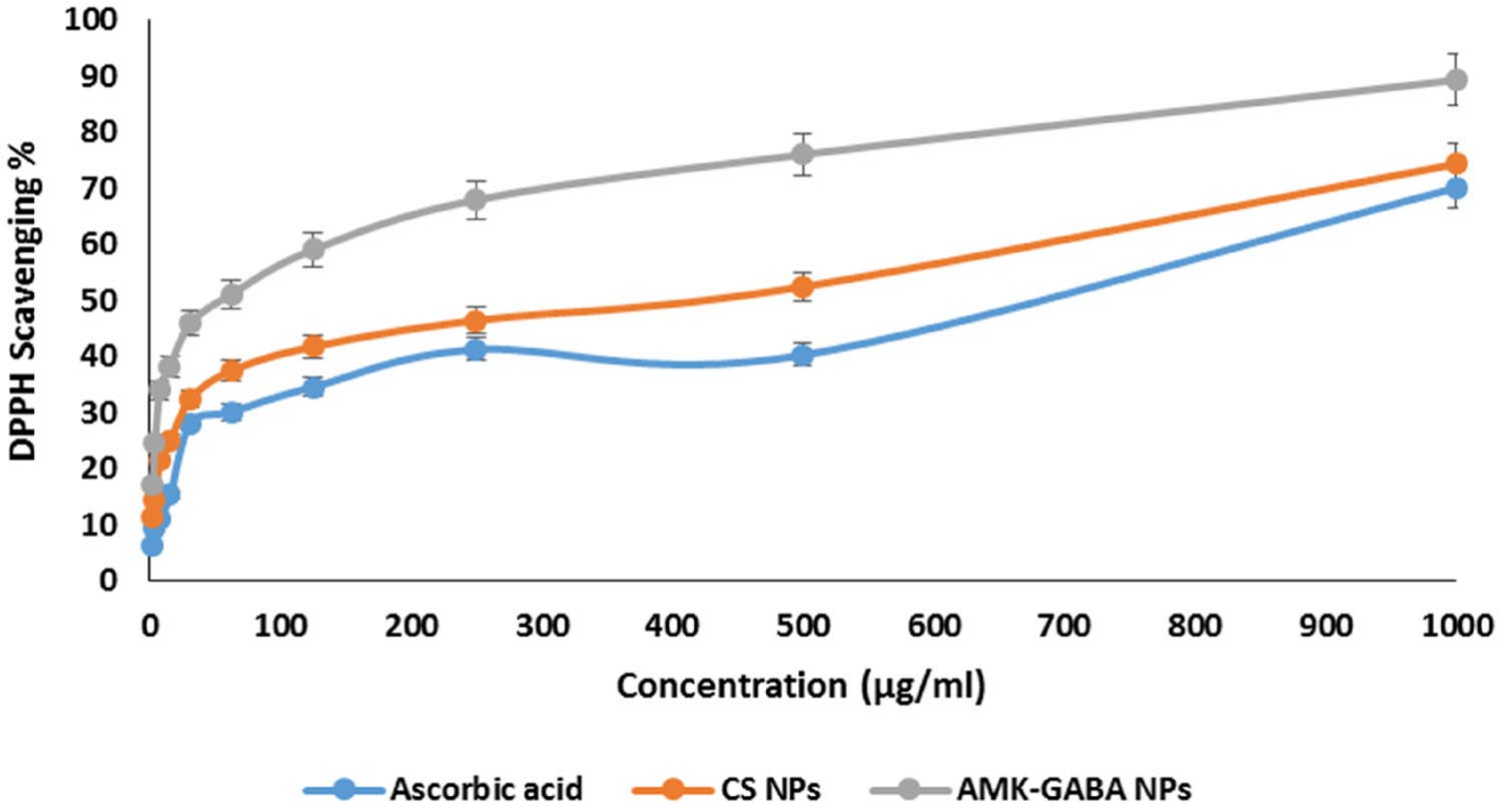
Antioxidant activity (DPPH scavenging assay) of chitosan nanoparticles (CSNPs) and amikacin-GABA chitosan nanoparticles (AMK–GABA–CSNPs) in comparison to ascorbic acid

DPPH was applied to test the antioxidant activity of the AMK–GABA–CSNPs. Figure 3 illustrates the scavenging activity of AMK–GABA–CSNPs using ascorbic acid as the reference antioxidant. The scavenging activity showed a dose-dependent pattern, increasing with an increase in dose of the AMK–GABA–CSNPs in the range 1.95–1000 g/ml, resulted in an improvement in percentage DPPH radical scavenging capacity. The AMK–GABA–CSNPs had a stronger scavenging potential than both ascorbic acid (as a control) and unloaded CSNPs. AMK–GABA–CSNPs with a concentration of 1000 g/ml had the highest DPPH scavenging activity (89.4%). This indicated that the synthesized AMK–GABA–CSNPs have promising antioxidant capabilities.

### The effect of AMK–GABA NPs on coagulation time

**Fig. 4:**
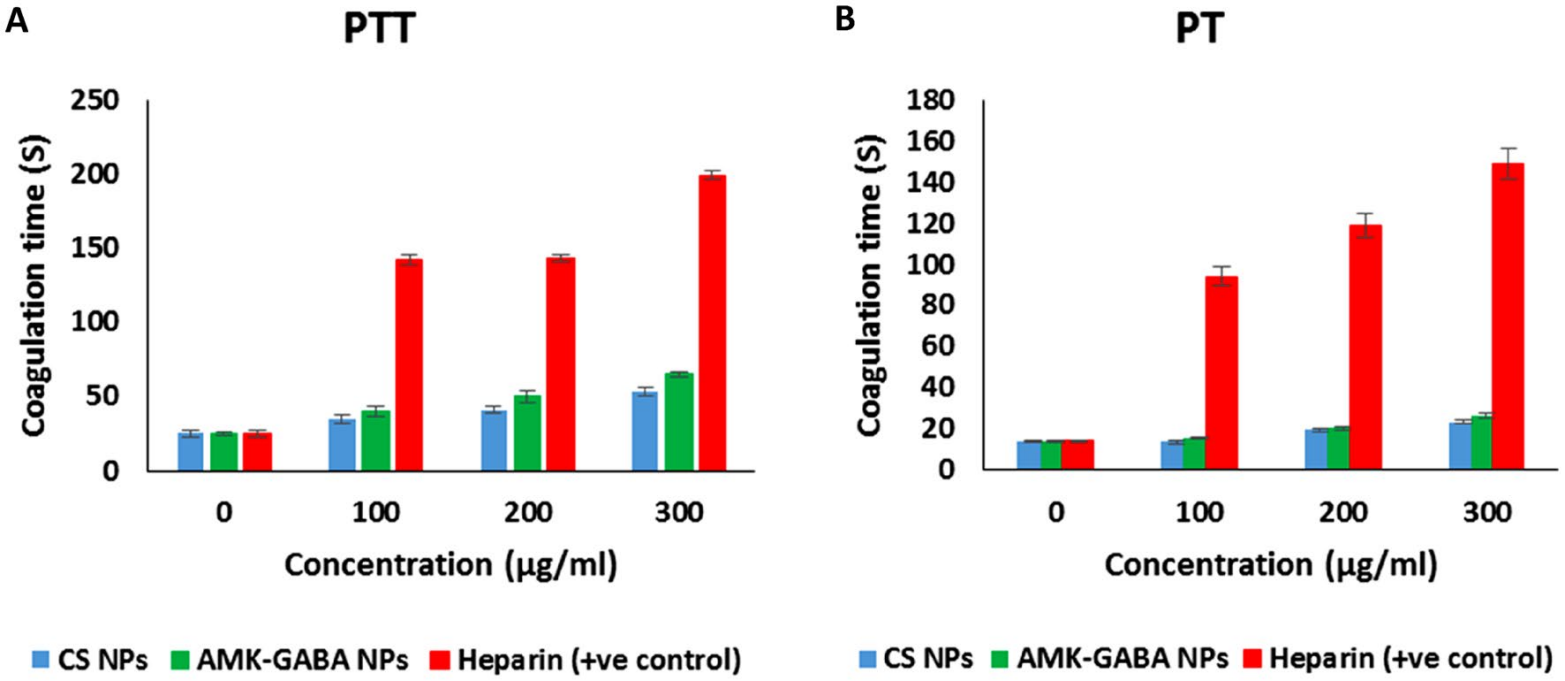
Effects of amikacin–GABA chitosan nanoparticles (AMK–GABA–CSNPs) and chitosan nanoparticles (CSNPs) on **A** prothrombin time (PT) and **B** partial thromboplastin time (PTT)

To evaluate the effect of AMK–GABA–CSNPs on plasma coagulation time, test samples at three concentrations (100, 200, and 300 g/mL) were incubated with rat plasma for 30 min. In this assay, both blood coagulation pathways, PT (Fig. 4A) and PTT (Fig. 4B), were separately tested. The results showed that AMK–GABA–CSNPs at the different concentrations had no significant effect on plasma coagulation for both intrinsic and extrinsic pathways compared to the untreated control. So, when suspended in saline, AMK–GABA–CSNPs had no effect on the coagulation time of rat blood.

### The effect of AMK–GABA–CSNPs on hemolytic activity

**Fig. 5:**
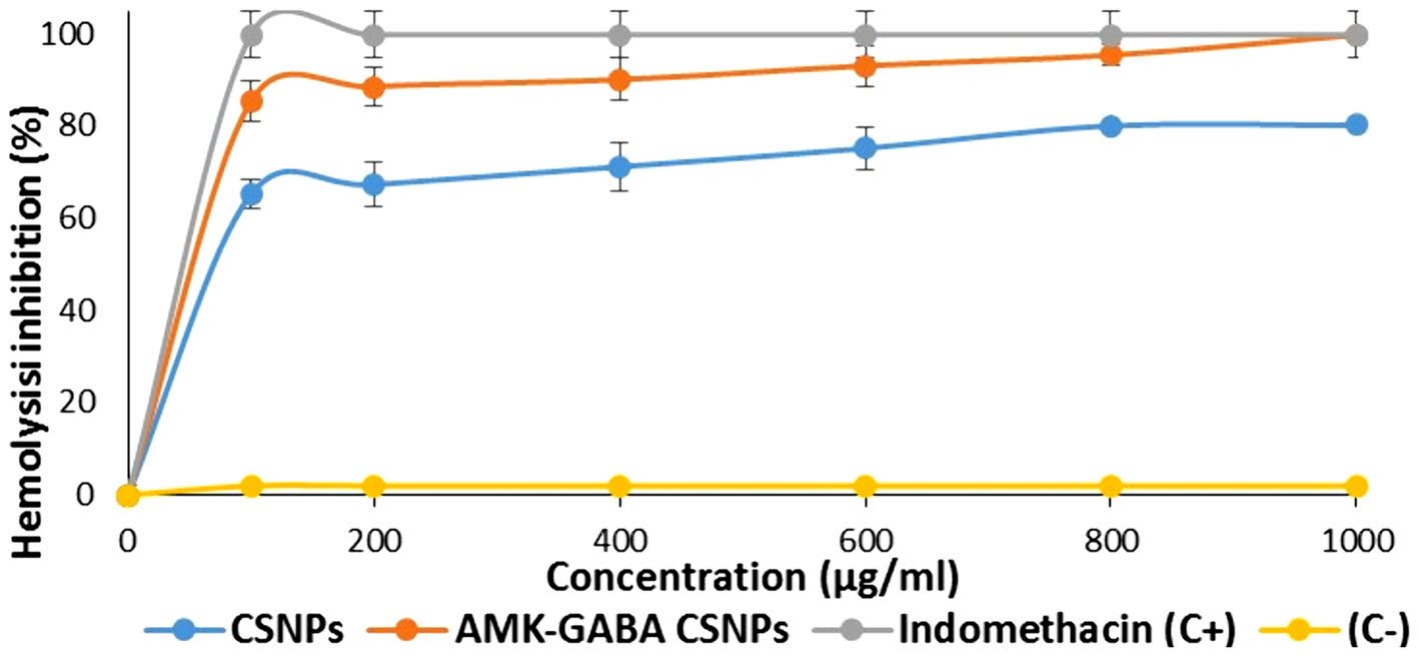
Hemolysis assay of chitosan nanoparticles (CSNPs) and amikacin–GABA chitosan nanoparticles (AMK–GABA–CSNPs). Indomethacin showed the highest hemolysis inhibition ability activity

As all the biomaterials reach the blood stream and come into contact with RBCs, AMK–GABA–CSNPs hemolytic activity was evaluated following a 1-h incubation at different concentrations with RBCs. Results showed that at concentrations of 100–1000 g/ml, AMK–GABA–CSNPs inhibited hemolysis by a percentage ranging from 85.41 to 99.81%, respectively (Fig. 5). On the other hand, the unloaded CSNPs reported significantly lower hemolysis inhibition percentages (65.21–80.21%) at the same concentrations.

### The cytotoxic effect of AMK–GABA–CSNPs on Caco-2 cell line

**Fig. 6:**
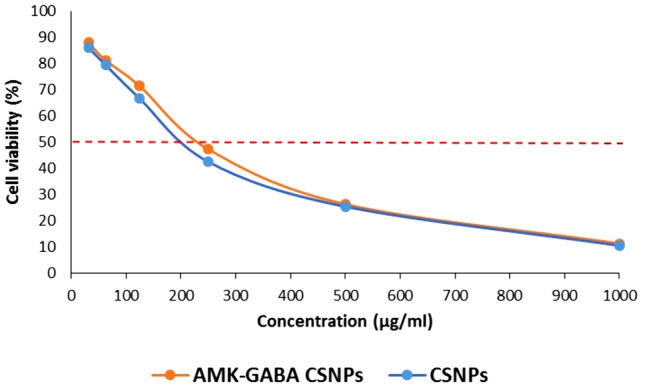
The in vitro cytotoxicity (MTT assay) of chitosan nanoparticles (CSNPs) and amikacin–GABA–chitosan nanoparticles (AMK–GABA–CSNPs). The dashed line indicates 50% cell viability

AMK–GABA–CSNPs toxicity was assessed by MTT compared to CSNPs as described previously for Caco-2, over a varied series of concentrations (31.25–1000 µg/ml) (Fig. 6). After 24h culture, the viability of Caco-2 cells was dependent on the concentration of the synthesized NPs. At the minimum dose used (31.25 µg/ml), the cell viability were 86.2% (CSNPs) and 88.1% (AMK–GABA–CSNPs), parallel to a cytotoxic activity of 13.8% and 11.9, respectively. At the maximum used dose (1000 µg/ml), the cell viability levels were 10.6% for CSNPs and 11.4% for AMK–GABA–CSNPs, parallel to a cytotoxic effect of 89.4% and 88.6%, respectively. AMK–GABA–CSNPs was not cytotoxic in a concentration range from 31.25 to 240.7 µg/ml, while AMK–GABA–CSNPs and CSNPs induced significant decreases in cell viability above 250 µg/ml (IC-50 of 240.7 µg/ml) with the maximal effect at 1000 µg/ml.

### The effects of unloaded AMK with GABA and AMK–GABA–CSNPs on kidney functions

**Fig. 7:**
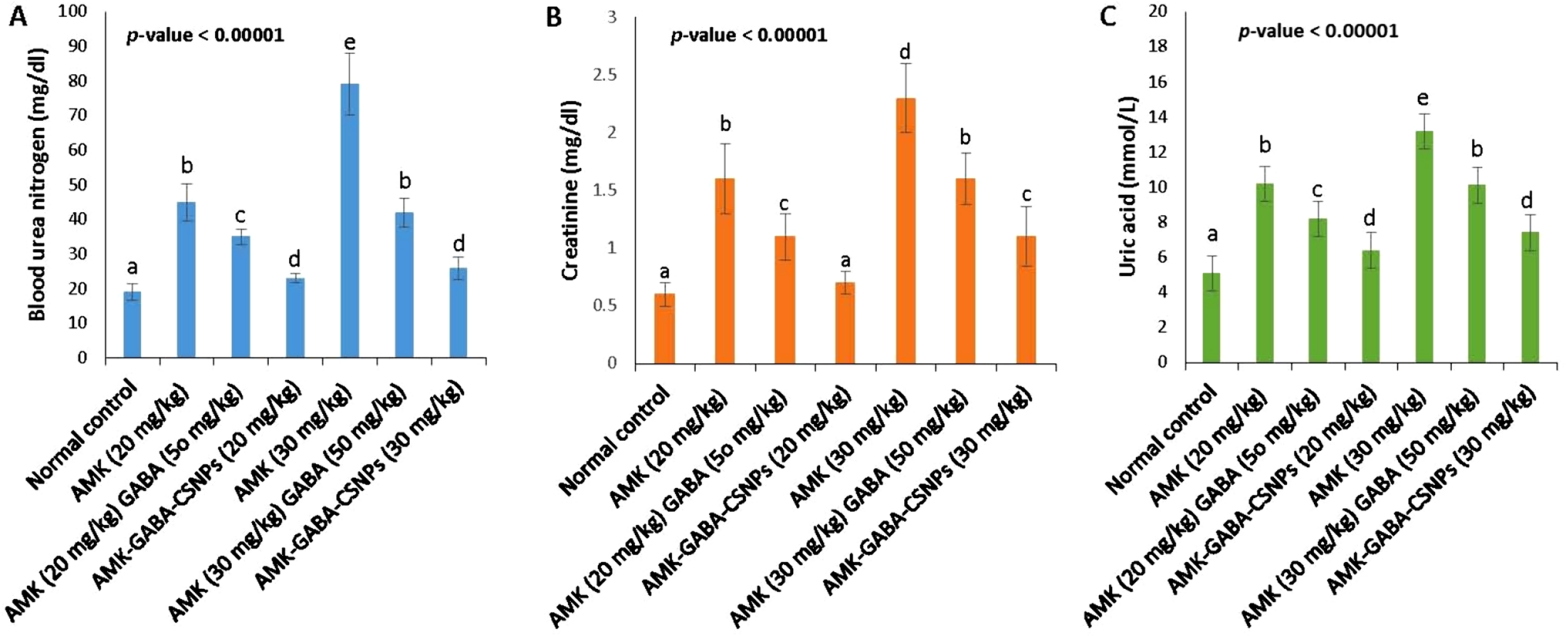
Effects of unloaded amikacin (AMK) with gamma amino butyric acid (GABA) and their combination loaded on chitosan nanoparticles (AMK–GABA–CSNPs) on **A** blood urea nitrogen (BUN), **B** serum creatinine (CRE) and **C** uric acid (UA) levels. Data were expressed as mean ± S.E (*n* = 6). Statistical analysis was carried out by one-way ANOVA followed by post hoc Tukeyʼs test. Values marked with the same letter are not significantly different (*p* ≥ 0.05), whereas those marked with different ones are significantly differed (*p* < 0.05)

The serum levels of BUN, CRE, and UA in comparison to the normal control are shown in Fig. 7. AMK (20 and 30 mg/kg) administration for 21 days resulted in a significant increase (*P* < 0.05) in serum BUN (45.21 ± 0.03 and 79.06 ± 0.11 mg/dl) (Fig. 7a), CRE (1.64 ± 0.31 and 2.38 ± 0.32 mg/dl) (Fig. 7b), and UA (10.25 ± 1.12 and 13.20 ± 1.27 mmol/l) (Fig. 7c), respectively. The injection of a high AMK dose (30 mg/kg) affected the serum levels of BUN, CRE, and UA more significantly than the 20 mg/kg dose. The simultaneous treatment of unloaded GABA (50 mg/kg) with each of 20 or 30 mg/kg AMK showed a significant decline (*P* < 0.05) in serum BUN (35.02 ± 2.2 and 42.04 ± 4.10 mg/dl, respectively), CRE (1.01 ± 0.17, and 1.62 ± 0.22 g/dl, respectively), and UA (8.24 ± 2.16 and 10.12 ± 0.98 mmol/l, respectively); compared to the corresponding nephrotoxic groups. AMK–GABA–CSNPs at either 20 or 30 mg/kg induced significant reductions of serum BUN (23.01 ± 1.41 and 26.21 ± 3.31 mg/dl), CRE (0.71 ± 0.11 and 1.1 ± 0.26 mg/dl) and UA (6.41 ± 1.39 and 7.4 ± 0.77 mmol/l). The reduction was significant (*P* < 0.05) compared to the corresponding unloaded AMK–GABA groups.

### The effects of unloaded AMK with GABA and AMK–GABA–CSNPs on renal MDA, SOD and GSH

**Fig. 8:**
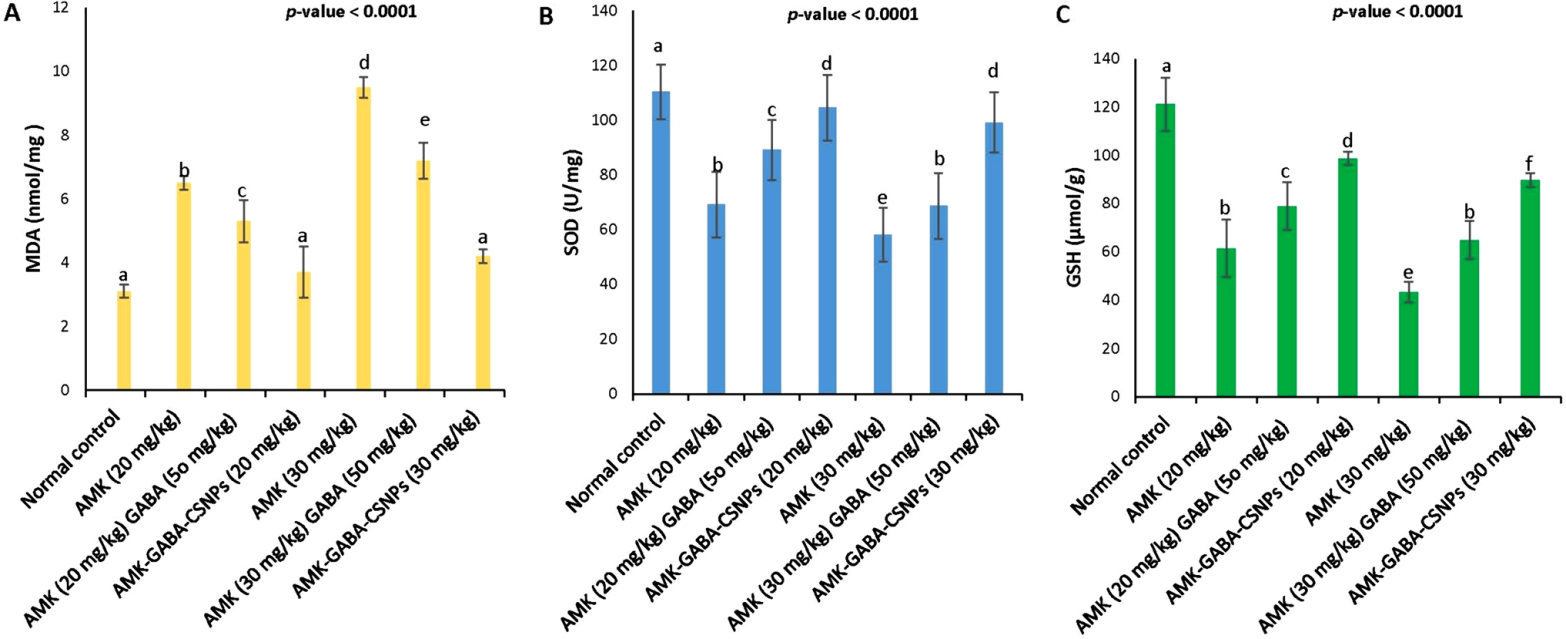
Effects of unloaded amikacin (AMK) with gamma amino butyric acid (GABA) and their combination loaded on chitosan nanoparticles (AMK–GABA–CSNPs) on **A** renal malondialdehyde (MDA) content, **B** superoxide dismutase (SOD) activity and **C** glutathione (GSH) content. Data were expressed as mean ± SE (*n*  =  6). Statistical analysis was carried out by one-way ANOVA followed by post hoc Tukeyʼs test. Values marked with the same letter are not significantly different (*p* ≥ 0.05), whereas those marked with different ones are significantly differed (*p* < 0.05)

Injection of AMK in both doses (20 and 30 mg/kg) significantly (*P* < 0.05) increased the MDA level (Fig. 8a) in renal homogenates (6.51 ± 0.22 and 9.53 ± 0.32 nmol/mg, respectively). Concurrent with this, the enzymatic antioxidant SOD (69.21 ± 12.03 and 58.22 ± 9.91 U/mg, respectively) (Fig. 8b) and GSH level (61.42 ± 12.05 and 43.25 ± 4.40 µmol/g), respectively) (Fig. 8c) were suppressed, compared to the normal control. The effect of AMK on MDA, GSH and SOD was significantly dose-dependent. Combined administration of unloaded AMK–GABA significantly (*P* < 0.05) reduced the elevated MDA levels in case of both 20 and 30 mg/kg AMK (5.11 ± 0.81 and 7.22 ± 0.56 nmol/mg, respectively). In addition to the significant MDA reduction, the antioxidant condition of the renal tissue was ameliorated via a significant (*P* < 0.05) elevation of SOD (89.23 ± 11.10 and 68.8 ± 12.71 U/mg, respectively) and GSH (78.9 ± 8.1 and 64.82 ± 7.97 µmol/g, respectively), compared to the corresponding nephrotoxic groups. In case of injection with AMK–GABA–CSNPs, surprising results were recorded as the levels of MDA (3.71 ± 0.81 and 4.22 ± 0.21 nmol/mg, respectively) and SOD (104.64 ± 12.03 and 99.51 ± 11.13 U/mg, respectively) were almost normalized with both low and high AMK dose formulations with a non-significant difference (*P* ≥ 0.05) form the normal control. Furthermore, GSH levels were significantly upgraded for both 20 and 30 mg/kg AMK (98.74 ± 2.71 and 89.72 ± 2.92 µmol/g, respectively).

### The effects of unloaded AMK with GABA and AMK–GABA–CSNPs on renal inflammatory cytokines

Inflammatory cytokines (IL-1β, IL-4, IL-6, IL-17, IL-18, TNF-α and IFN-γ) were measured in renal homogenates from the different experimental groups, and the results are recorded in Table 1. Compared to the control group, free AMK injection (20 and 30 mg/kg) induced significant (*P* < 0.05) elevation of all the monitored renal cytokines. The elevation was dose-dependent and significantly (*P* < 0.05) higher in AMK (30 mg/kg) than AMK (20 mg/kg) group. Unloaded AMK–GABA combination developed a significant (*P* < 0.05) decrease in the inflammatory cytokines. In case of injection with AMK–GABA–CSNPs either 20 or 30 mg/kg, the improvement in inflammatory response was more significant (*P* < 0.05) compared to the corresponding unloaded AMK–GABA combination groups.

**Table 1:** Effects of unloaded amikacin (AMK) with gamma amino butyric acid (GABA) and their combination loaded on chitosan nanoparticles (AMK–GABA–CSNPs) on renal levels of interleukin (IL) 1 beta (1β), IL-4, IL-6, IL-17, IL-18, tumor necrosis factor alpha (TNF-α) and Interferon gamma (IFN-γ)

	IL-1β (pg/g)	IL-4 (pg/g)	IL-6 (pg/g)	IL-17 (pg/g)	IL-18 (pg/g)	TNF-α (pg/g)	IFN-γ (pg/g)
Normal control	77.8 ± 1.3^a^	215.4 ± 3.5^a^	191.2 ± 11.1^a^	630.2 ± 7.9^a^	113.5 ± 2.4^a^	615.4 ± 6.4^a^	25.5 ± 4.2^a^
AMK (20 mg/kg)	123.5 ± 3.4^b^	458.2 ± 2.5^b^	546.1 ± 8.4^b^	840.2 ± 4.8^b^	198.4 ± 5.2^b^	940.2 ± 7.8^b^	90.2 ± 5.1^b^
AMK (20 mg/kg) + GABA (50 mg/kg)	101.9 ± 5.8^c^	333.4 ± 4.6^c^	401.2 ± 3.5^c^	798.2 ± 5.7^c^	172.3 ± 3.2^c^	815.2 ± 5.6^c^	77.2 ± 3.4^c^
AMK–GABA–CSNPs (20 mg/kg)	80.1 ± 2.2^a^	220.1 ± 2.2^a^	201.1 ± 3.7^a^	645.3 ± 5.8^a^	128.4 ± 4.4^a^	701.2 ± 3.2^d^	44.2 ± 1.4^d^
AMK (30 mg/kg)	167.5 ± 3.1^d^	529.2 ± 2.9^d^	680.2 ± 8.1^d^	1120.4 ± 9.4^d^	222.4 ± 2.6^d^	1021.1 ± 8.5^e^	115.6 ± 3.9^e^
AMK (30 mg/kg) + GABA (50 mg/kg)	157.9 ± 7.6^e^	401.2 ± 7.5^e^	498.2 ± 4.5^e^	980.2 ± 5.7^e^	209.1 ± 9.5^b^	899.4 ± 11.4^f^	91.2 ± 3.5^b^
AMK–GABA–CSNPs (30 mg/kg)	117.8 ± 3.4^b^	312.5 ± 3.9^c^	294.4 ± 6.8^f^	750.2 ± 3.9^f^	134.5 ± 5.4a^a^	790.1 ± 7.3^g^	55.2 ± 2.1^d^

Data were expressed as mean ± S.E (*n* = 6). Statistical analysis was carried out by one-way ANOVA followed by post hoc Tukeyʼs test. In each column, values superscripted with the same letter are not significantly different (*p* ≥ 0.05), whereas those marked with different ones are significantly differed (*p* < 0.05)

### The effects of unloaded AMK with GABA and AMK–GABA–CSNPs on renal histopathology and fibrosis

**Fig. 9:**
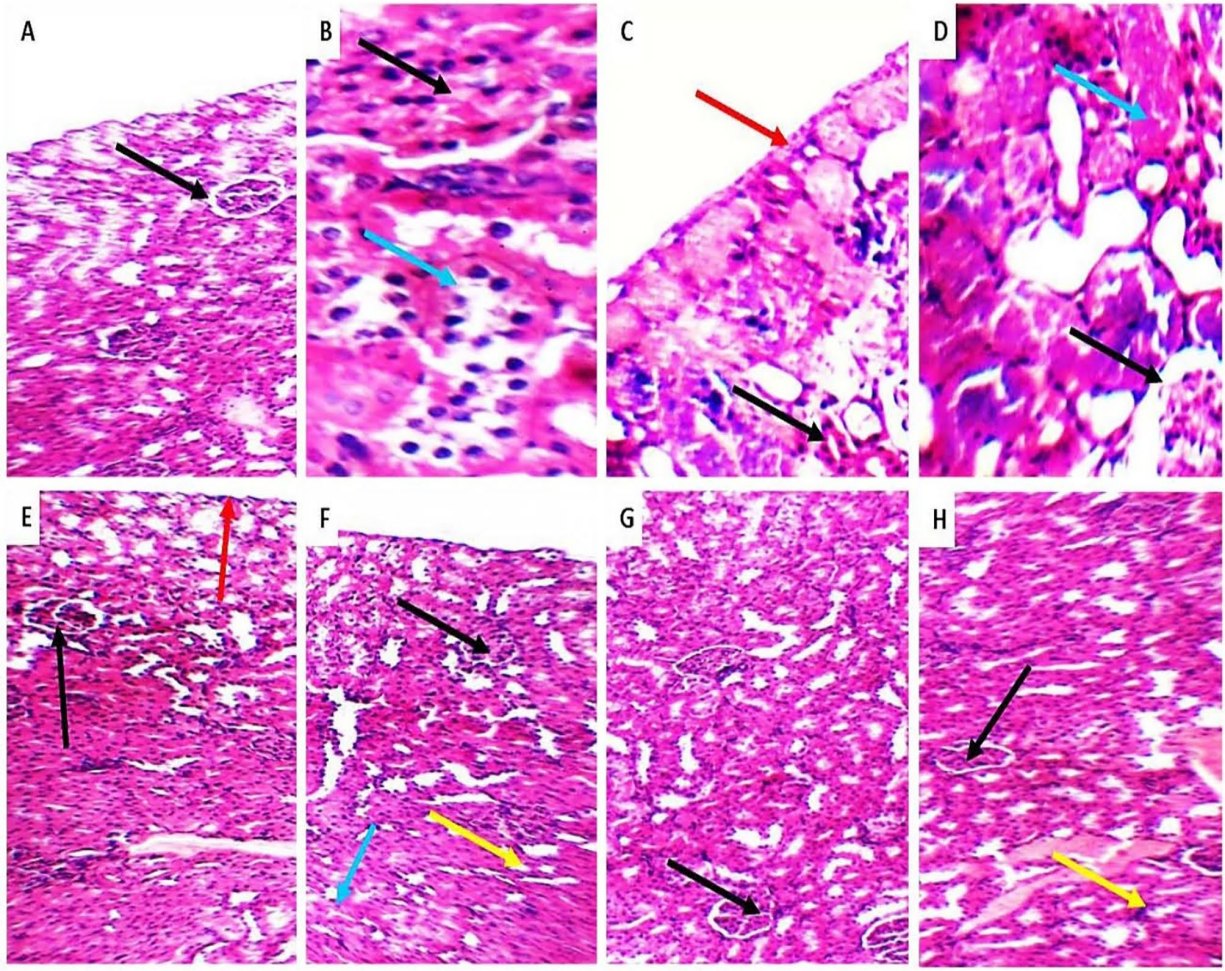
Photomicrographs of renal tissue sections stained with haematoxylin and eosin. **A** Average renal capsule (black arrow), average glomeruli, and average tubules in normal control group (200×); **B** Mildly congested glomeruli (black arrow) with average Bowman’s space, proximal tubules with mildly edematous epithelial lining (blue arrow), and average interstitium in group receiving 20 mg/kg AMK (400×); **C** and **D** Average renal capsule (red arrow), scattered small-sized glomeruli (black arrow), and marked tubular necrosis (blue arrow) in group receiving 30 mg/kg AMK (200×); **E** Average renal capsule (red arrow), few scattered small-sized glomeruli (black arrows), and average tubules in group that received unloaded AMK (20 mg/kg/day; i.p.) and GABA (50 mg/kg/day) ( 200×); **F** Scattered small-sized glomeruli (black arrow), markedly congested blood vessels (blue arrow), and interstitial inflammatory infiltrate (yellow arrow) in group that received unloaded AMK (30×mg/kg/day; i.p.) and GABA (50 mg/kg/day; i.p.) ( 200×); **G** Average renal capsule, few scattered small-sized glomeruli (black arrow), and average tubules in group that received AMK–GABA–CSNPs (20 mg/kg/day; i.p.) ( 200×); **H** Average glomeruli (black arrows), and mildly congested blood vessels (yellow arrow) (200×) in group receiving AMK–GABA–CSNPs (30 mg/kg/day; i.p.)

AMK (20 or 30 mg/kg) induced marked alterations in normal kidney construction; small-sized congested glomeruli, considerably edematous epithelial lining, tubular necrosis, congested blood vessels and inflammatory cell infiltrates. The injection of AMK–GABA combinations alleviated nephrotoxicity by diminishing the percentage of inflammation and necrosis. Administration AMK–GABA-NPs (20 or 30 mg/kg) showed maximal reduction in inflammation and necrosis compared to AMK–GABA combinations (Fig. 9).

**Fig. 10:**
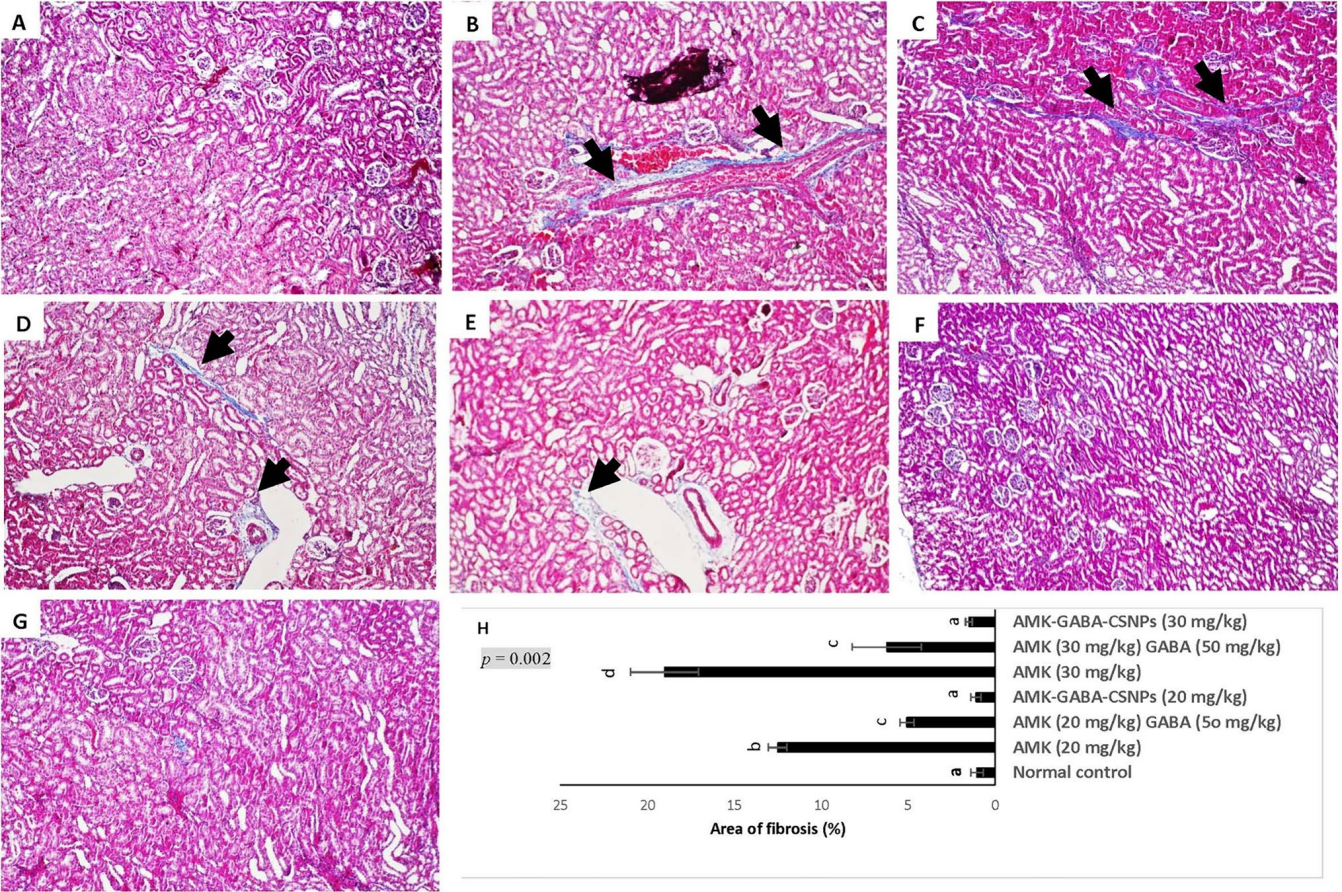
Photomicrographs of kidney tissue sections stained with Masson’s trichrome (100×). **A** Normal control rat presenting absence fibrosis or collagen deposition; **B** AMK (20 mg/kg); and **C** AMK (30 mg/kg) nephrotoxic groups: displaying central fibrosis and blue stained collagen between glomeruli and tubules at the corticomedullary portion (black arrow); **D** AMK (20 mg/kg) + GABA (50 mg/kg); and **E** AMK (30 mg/kg) + GABA (50 mg/kg) combination groups showing decline in fibrosis with minimal stained collagen accumulation (black arrow); **F** AMK–GABA–CSNPs (20 mg/kg) or **G** (30 mg/kg) confirmed complete withdrawal of collagen and fibrosis. **H** The area of fibrosis for each experimental group was analyzed via Image Pro Plus (IPP) 6.0 software in ten randomly examined fields from each section. Mann–Whitney U test was applied for significance between groups. Values marked with the same letter are not significantly different (*p*  ≥ 0.05), whereas those marked with different ones are significantly differed (*p* < 0.05)

Using Masson's trichrome stain, the extent of fibrosis and collagen proliferation in renal tissues was assessed after 3 weeks in distinct experimental groups. The AMK-treated (20 or 30 mg/kg) groups showed deposits of collagen around the glomeruli, tubules, and perivascular region. Semi-quantitative analysis indicated that the area of collagen deposition in the AMK-treated (20 or 30 mg/kg) groups (Fig. 10B, C) was considerably greater (*P* < 0.05) than in the normal control (Fig. 10A). A significant decrease of (*P* < 0.05) collagen area deposition in AMK (20 mg/kg) + GABA (50 mg/kg) (Fig. 10D) and AMK (30 mg/kg) + GABA (50 mg/kg) (Fig. 10E) with no fibrosis and minimal stained collagen deposition. AMK–GABA–CSNPs (20 mg/kg) (Fig. 10F) or (30 mg/kg) (Fig. 10G) demonstrated almost complete disappearance of collagen and fibrosis. The area of fibrosis for each experimental group was quantified in Fig. 10H.

## Discussion

AMK-induced nephrotoxicity is mainly caused by oxidative injury due to renal cortical lipoperoxidation and peroxide generation (Kaynar et al. 2007). As the oxidative injury is one of the primary causal factors relating to AMK-induced renal injury. The current work attempts to diminish the oxidative injury via co-administration of GABA, a powerful antioxidant, with AMK loaded on CSNPs. CSNPs can resolve the majority of the initial problems with drug delivery, including fewer side effects, poor bioavailability, accurate distribution and slow release to the site of action resulting in excellent pharmacological efficiency and a therapeutic outcome (Mikušová and Mikuš 2021).

There have been several reports of AMK nanoparticles with various structures. AMK was encapsulated using gold nanostars (GNS) (Aguilera-Correa et al. 2022). Poly (lactic acid-co-polyethylene glycol) (PLA-PEG), low-molecular-weight poly (lactic acid) (PLA), and with poly (vinyl alcohol) (PVA) were also applied in AMK NPs (Glinka et al. 2021). Reddy and Damodharan (2022) also optimized AMK CSNPs with a double efficacy as pure AMK. Rahmati et al. (2022) confirmed the synthesis of AMK-loaded niosome nanoparticles with enhanced antibacterial activity. To the best of our knowledge, the present study is the first to successfully prepare AMK–GABA–CSNPs by the ionic gelation method. We applied this procedure as it is the most frequently used technique to develop CSNPs. In addition to being easy to utilize, this technique does not alter the drug’s chemical structure (Hoang et al. 2022). Shilpa et al. (2012) suggested the formation of GABA–CSNPs by bond formation between the amino group of CS and the carboxyl group of GABA. The particle size was 77.5 ± 16.5 nm. So that the AMK–GABA–CSNPs synthesized here were within the range of 1 to 100 nm, which defines the nanoparticle scale in accordance with the Standard Terminology Relating to Nanotechnology E 2456-06 (ASTM 2006). This range is smaller than the size range recorded in a previous study by Reddy and Damodharan (2022), whose particles presented 168–297 nm when formulated as AMK CSNPs. This finding suggests that nanoparticle suspension preparation standards should be taken into consideration. The ZP was + 38.94 ± 2.65 mV. The positively charged amine group of the main component, chitosan, is the cause of the NPs’ positive character. The formed AMK–GABA–CSNPs’ positive zeta potential reflects the higher stability of the nanoparticles used in the formulation. Reddy and Damodharan (2022) synthesized AMK CSNPs with ZP range from +34.65 mV to +43.24 mV. Ghaffari et al. (2010) also recorded positive ZP ranging from +4 mV to +16 mV after preparation of AMK-loaded solid lipid nanoparticles. In addition, the FTIR results of AMK–GABA–CSNPs showed that GABA and AMK were effectively loaded into the CSNPs. The resulting spectra for CS, AMK and GABA were in agreement with that obtained in earlier reports (Suresh et al. 2008; Zareie et al. 2019; Abdel-Hakeem et al. 2022).

An efficient antioxidant possesses reducing power due to its capacity to transfer electrons. The synthesized AMK–GABA–CSNPs had significantly higher antioxidant activity compared to CSNPs and ascorbic acid as control. The reducing power of the AMK–GABA–CSNPs can be due to the large number of hydrogen ions produced from GABA molecules. Falah et al. (2021) also suggested a significant antioxidant efficiency of GABA via its ability to neutralize DPPH radicals. According to Liu et al. (2016), enzymatic hydrolysis and decarboxylation of GABA cause the amino acids to split apart and release tyrosine amino acids in the terminal C peptide, which become capable of donating electrons and stopping the free-radical chain reaction.

When applying biomaterials, effective blood coagulation must be taken into consideration. As hemorrhage and thrombosis may result from an imbalance between pro- and anti-coagulation activities. This study used PT and PTT assays to determine how AMK–GABA–CSNPs affected blood hemostasis. Extrinsic and intrinsic blood coagulation pathways are represented by PT and PTT, respectively. In the present study, the formulated AMK–GABA–CSNPs showed no significant effectiveness in accelerating blood coagulation time (PT and PTT), indicating no interruption of the thromboembolism in the vascular system. Earlier studies suggested the interaction of the positively charged amino group along the CS molecule with the negatively charged erythrocytes and platelets to change the microstructure of hemoglobin and make blood more viscous (and prolong PT and PTT) (de Lima et al. 2015; Wang et al. 2019, 2021). However, the formation of a bond between the amino group of CS and the carboxyl group of GABA may explain the obtained results of normal PT and PTT for AMK–GABA–CSNPs. In consonance with our findings, Tyurenkov et al. (2014) reported in vivo improvement of the hemostasis system with GABA derivatives administration via changes in calcium levels and calmodulin activity in platelets. In addition, Jaccob et al. (2019) suggested that AMK had no apparent impact on the PTT clotting time and that the only way it could extend PT was at high concentrations, as it could prevent the production and activation of fibrinogen and inhibit the endogenous clotting factor in order to prevent platelets from aggregating.

Hemolytic activity was carried out to assess the safety of the produced AMK–GABA–CSNPs. Considering the results obtained, it can be stated that even at high concentrations AMK–GABA–CSNPs have no hemolytic effect and are appropriate for systemic administrations with promising in vitro anti-inflammatory activity. In agreement with the present results, Abdel-Hakeem et al. (2022) described erythrocytes as osmometers that lyse in response to changes in the blood’s osmotic and physical parameters and confirmed that gentamicin–ascorbic acid CSNPs did not exhibit hemolytic activity.

The MTT assay measures mitochondrial activity, which is directly related to cell viability Dead cells cannot change the MTT tetrazolium salt to colored formazan crystals, whereas metabolically active cells can. The results of the MTT experiments supported AMK–GABA–CSNPs' good cell compatibility, as there is no evidence of cytotoxicity and well tolerated by cells up to concentration of 240.7 µg/ml.

In the present investigation, we established an AMK-induced acute nephrotoxicity model using Sprague–Dawley rats and verified that AMK injection at 20 and 30 mg/kg aggravated symptoms of renal injury after 3 weeks. Elevated levels of BUN, CRE and UA indicated renal dysfunction (Gounden et al. 2020). The dose-dependent elevations of BUN, CRE and UA due to AMK injection were consistent with previous reports (Batoo et al. 2018; Madbouly et al. 2021; Azırak 2023). Whereas unloaded AMK–GABA combination had recoded a protective effect as it suppressed serum levels of BUN, CRE and UA. In agreement with our findings, Lee et al. (2022) suggested GABA protective effect against cisplatin-induced nephrotoxicity via inhibition of tubular dilation and hemorrhage. Interestingly, AMK–GABA–CSNPs injections (20 or 30 mg/kg) recorded amelioration of kidney functions with higher significance compared to unloaded AMK–GABA combinations. Shilpa et al. (2012) attributed this to chitosan's positive charge, high cell binding affinity, and the prolonged GABA effect of GABA when loaded on CS due to lower exposure to the internal body environment.

It has been proposed that AMK-induced nephrotoxicity was triggered due to the production of highly destructive free radicals like superoxide anions, H_2_O_2_, and hydroxyl radicals (Batoo et al. 2018). In harmony with this theory, our findings demonstrated AMK injection markedly increased lipid peroxidation (LPO), indicated by higher MDA, and inhibited GSH and SOD’s antioxidant activity in renal homogenates, making the kidneys more susceptible to oxygen radical stress.

The intraperitoneal injection of 50 mg/kg GABA with AMK (20 or 30 mg/kg) potentiated the antioxidant activity of SOD and GSH and suppressed MDA. This demonstrates that GABA can defend against AMK-induced nephrotoxicity. In agreement with our results, Ali et al. (2015) reported the ameliorative action of oral GABA on SOD and GSH and protection against cisplatin-induced nephrotoxicity through increasing its urinary excretion, with no effect on the drug's therapeutic efficacy by boosting its urine excretion. Furthermore, Sasaki et al. (2006) reported the protective action of GABA against oxidative stress during chronic renal failure caused by nephrectomy. In comparison to the unloaded AMK–GABA combination, AMK–GABA–CSNPs demonstrated a significantly enhanced renal antioxidant system as it almost normalized MDA and SOD levels with a significant increase of GSH activity. These findings suggest that the loading on CSNPs benefits the antioxidant properties of GABA. This may be attributed to the improved solubility and bioavailability of GABA after loading. In addition, CSNPs’s synergistic effect contributes high antioxidant activity and free radical scavenging capacity, which can reduce lipid peroxidation (MDA levels) and boost the antioxidant defense system, protecting renal tissue from free radical damage induced by AMK (Samadarsi and Dutta 2020; Halawa et al. 2022).

As the pathogenesis of acute renal injury is believed to be seriously influenced by inflammation, the present study monitored the inflammatory immune response. Because of direct contact with reactive oxygen species (ROS), renal vascular endothelial cells and tubular epithelium were involved in the early initiation and extension of inflammatory responses in the injured kidney. This is done via the release of various local inflammatory cytokines such as IL-1β, IL-4, IL-6, IL-17, IL-18, TNF-α and IFN-γ. The injured kidney’s proximal tubules, lymphocytes, neutrophils, and macrophages develop IL-18, a proinflammatory cytokine. Caspase-1 activates IL-18 which induces the synthesis of many cytokines and chemokines that trigger activation of T helper cells and lymphocyte proliferation (Akcay et al. 2009). The accumulation of ROS in renal tissue activates nuclear factor kappa B (NF-kB), triggering the inflammatory signaling cascade and express TNF-α. TNF-α enhances the production of other inflammatory cytokines, especially IFN-γ, IL-1β and IL-6 which are powerful proinflammatory cytokines that play a role in inflammatory renal damage (Kumar et al. 2015; Tripathi and Alshahrani 2021; Farid et al. 2023). IL-17 is a proinflammatory cytokine that is primarily produced by T helper 17 (Th17) a subset of CD4 + T cell. A broad spectrum of factors, including cytokines like IL-6 and IL-1β, influence the differentiation of CD4 + cells to Th17 cells (Wang et al. 2023). IL-17 is elevated in blood and renal lysate in case of nephrotoxicity (Collett et al. 2022). The actual role of IL-4 during renal injury is complex and contrasting to be described. As some reports have found IL-4 to play a significant role in enhancing the recovery of tubular damage in renal disease by inducing M2 macrophage phenotypic polarization (Zhang et al. 2017). On the other hand, other reports suggest that the IL-4 receptor α chain/STAT6 pathway promotes fibrosis and renal disease progression (Liang et al. 2017). The present work recorded a significant increase in renal levels of IL-1β, IL-4, IL-6, IL-17, IL-18, TNF-α and IFN-γ that may potentiate AMK-induced nephrotoxicity. These cytokines were reported to elevate due to drug-induced nephrotoxicity (Saeed et al 2022; Chen et al. 2022; Dari et al. 2023). In this investigation, we discovered that the unloaded AMK–GABA combination dropped the renal levels of IL-1β, IL-4, IL-6, IL-17, IL-18, TNF-α and IFN-γ in correlation with AMK dose. The capacity of unloaded AMK–GABA combination to reduce inflammation and alleviate oxidative stress may be connected. GABA exerts its potent anti-inflammatory effects by inhibiting numerous signaling pathways that contribute to the generation of proinflammatory cytokines. As previously reported, one of these effects is that GABA is a paracrine and autocrine signaling molecule that activates GABA receptors on immune cells. GABA modulates immune function via attaching to GABA_A_ receptors, which modify proliferation and suppress the release of numerous cytokines (about 47 cytokines) (Bhandage et al. 2018). Besides, GABA_A_ receptor engagement is a powerful inhibitor of NF-kB activation by the kidneys' intracellular ROS generation after AMK therapy. As a result, the observed decrease in TNF-α, IL-1, and IL-6 levels following GABA therapy can be explained (Zhang et al. 2022). In agreement with our results, early studies revealed that GABA alter both innate and adaptive immunity as it lower Th17, CD4 + T cells and CD8 + T cells, promoted CD4 + and CD8 + regulatory T cell (Treg) responses and shift natural killers (NKs), dendritic cells (DCs), and macrophages toward anti-inflammatory phenotypes (Tian et al. 2014, 2019; Bhandage et al. 2020; Zhang et al. 2021).

Since previous investigations proved that CSNPs have various anti-inflammatory properties (Kim et al. 2004; Friedman et al. 2013; Jhundoo et al. 2020), we exactly studied whether the AMK-induced inflammatory cytokines could be regulated in the presence of GABA–CSNPs. Interestingly, we found that AMK–GABA–CSNPs minimized the renal inflammatory cytokines levels in a manner that was more significant than that of unloaded AMK–GABA combinations. The augmented anti-inflammatory activity detected for AMK–GABA–CSNPs may be attributed to the combining of the anti-inflammatory properties of GABA with CSNPs' intrinsic anti-inflammatory action in the same delivery platform. Similar to our study results, Liu et al. (2020) and Mohyuddin et al. (2021) revealed down-regulation of plasma levels of TNF-α, IL-1β, IL-6 and IL-10 in mice receiving the oral administration of CS. Yang et al. (2016) stated that CS suppresses the manifestation of inflammatory genes by inhibiting TLR4/NF-kB signaling pathways. Nomier et al. (2022) outlined that CSNPs provided considerable protection and improved CCl4-induced nephrotoxicity by lowering TNF-α and IL-1β.

According to the histopathology studies, AMK injection (20 or 30 mg/kg) was related to changes in the renal histological construction, particularly infiltration of inflammatory cell, degradation of tubular epithelial lining, and tubular necrosis. These findings corroborate recent findings (Madbouly et al. 2021; Saeed et al 2022) that AMK causes significant degenerative alterations in the kidney due to the accumulation of inflammatory mediators and ROS in the renal cortex. Unloaded AMK–GABA combinations decreased the histopathological alterations by lowering inflammation, necrosis, and fibrosis. These findings are comparable with that of Lee et al. (2022), who found that GABA significantly improved histological symptoms of nephrotoxicity such as tubular dilatation, renal hypertrophy, hemorrhage, and collagen deposition in cisplatin-induced nephrotoxicity. The anti-fibrogenic properties of GABA may be attributed to its antioxidant properties and the lowering of proinflammatory cytokines. Sasaki et al. (2007) revealed that GABA inhibits transforming growth factor-beta (TGF-β1) and fibronectin expression in renal tubules by binding to GABA_A_ and GABA_B_ receptors, which in turn reduce renal fibrosis. The administration of AMK–GABA–CSNPs revealed synergistic anti-inflammatory and anti-fibrogenic effects. In agreement with our findings, Qiao et al. (2014) illustrated the ability of CS-based nanocomplex to enhance specific renal targeting and could be used to design drug delivery systems that reduce renal fibrosis. Wu et al. (2022) suggested that CS exert excellent inhibitory effects on fibronectin, collagen deposition, and TGF-β1/Smad signal pathway.

## Conclusion

AMK–GABA–CSNPs were prepared, for the first time, via the ionic gelation method, and displayed spherical nano-sized particles of homogenous distribution. The synthesized NPs were evaluated in vitro and in vivo and showed antioxidant, anticoagulant and anti-hemolytic activities. AMK–GABA–CSNPs (20 and 30 mg/kg) improved renal functions compared to AMK-induced nephrotoxicity in rat model. Overall, the potential underlying mechanisms of the AMK–GABA–CSNPs to protect renal tissue were by suppressing oxidative stress, inhibition of inflammatory cytokines and reduction of collagen deposition. In comparison to the unloaded AMK–GABA combinations, AMK–GABA–CSNPs formulation was significantly more effective especially in low-dose group (20 mg/kg). Hence, AMK–GABA–CSNPs could afford a potential nano-therapeutic formula for the management of AMK-nephrotoxicity.

## Data Availability

All data generated or analyzed during this study are included in this published article.
